# Identification of small molecule allosteric modulators of 5,10-methylenetetrahydrofolate reductase (MTHFR) by targeting its unique regulatory domain

**DOI:** 10.1016/j.biochi.2021.01.007

**Published:** 2021-04

**Authors:** Gustavo A. Bezerra, Alexander Holenstein, William R. Foster, Bing Xie, Kevin G. Hicks, Céline Bürer, Seraina Lutz, Ayan Mukherjee, Dipika Sarkar, Debomita Bhattacharya, Jared Rutter, Arindam Talukdar, Peter J. Brown, Minkui Luo, Lei Shi, D. Sean Froese, Wyatt W. Yue

**Affiliations:** a Structural Genomics Consortium, Nuffield Department of Clinical Medicine, University of Oxford, OX3 7DQ, UK; b Division of Metabolism and Children's Research Center, University Children's Hospital Zürich, University of Zürich, Switzerland; c Computational Chemistry and Molecular Biophysics Unit, National Institute on Drug Abuse - Intramural Research Program, National Institutes of Health, Baltimore, USA; d Department of Biochemistry, University of Utah School of Medicine, USA; e Department of Organic and Medicinal Chemistry, CSIR-Indian Institute of Chemical Biology, 4 Raja S. C. Mullick Road, Kolkata, 700032, WB, India; f Structural Genomics Consortium, University of Toronto, Toronto, Ontario, M5G 1L7, Canada; g Chemical Biology Program, Memorial Sloan Kettering Cancer Center, New York, NY, USA; h Program of Pharmacology, Weill Cornell Medical College of Cornell University, New York, NY, USA

**Keywords:** 5,10-Methylenetetrahydrofolate reductase, One-carbon metabolism, Drug-development, Enzymatic inhibition, Small molecules

## Abstract

The folate and methionine cycles, constituting one-carbon metabolism, are critical pathways for cell survival. Intersecting these two cycles, 5,10-methylenetetrahydrofolate reductase (MTHFR) directs one-carbon units from the folate to methionine cycle, to be exclusively used for methionine and *S*-adenosylmethionine (AdoMet) synthesis. MTHFR deficiency and upregulation result in diverse disease states, rendering it an attractive drug target. The activity of MTHFR is inhibited by the binding of AdoMet to an allosteric regulatory domain distal to the enzyme’s active site, which we have previously identified to constitute a novel fold with a druggable pocket. Here, we screened 162 AdoMet mimetics using differential scanning fluorimetry, and identified 4 compounds that stabilized this regulatory domain. Three compounds were sinefungin analogues, closely related to AdoMet and *S*-adenosylhomocysteine (AdoHcy). The strongest thermal stabilisation was provided by (*S*)-SKI-72, a potent inhibitor originally developed for protein arginine methyltransferase 4 (PRMT4). Using surface plasmon resonance, we confirmed that (*S*)-SKI-72 binds MTHFR via its allosteric domain with nanomolar affinity. Assay of MTHFR activity in the presence of (*S*)-SKI-72 demonstrates inhibition of purified enzyme with sub-micromolar potency and endogenous MTHFR from HEK293 cell lysate in the low micromolar range, both of which are lower than AdoMet. Nevertheless, unlike AdoMet, (*S*)-SKI-72 is unable to completely abolish MTHFR activity, even at very high concentrations. Combining binding assays, kinetic characterization and compound docking, this work indicates the regulatory domain of MTHFR can be targeted by small molecules and presents (*S*)-SKI-72 as an excellent candidate for development of MTHFR inhibitors.

## 1 Introduction

5,10-Methylenetetrahydrofolate reductase (MTHFR) is the key enzyme in one-carbon metabolism (OCM) that connects the folate and methionine cycles. It catalyses the physiologically irreversible reduction of 5,10-methylenetetrahydrofolate (CH_2_-THF), derived in the folate cycle, to form 5-methyltetrahydrofolate (CH_3_-THF), requiring FAD as cofactor and NADPH as electron donor. Since the product CH_3_-THF is exclusively used in the methionine cycle by vitamin B_12_-dependent methionine synthase, the MTHFR reaction commits folate-carried one-carbon units towards methionine and *S*-adenosylmethionine (AdoMet) synthesis.

**Fig. 1:**
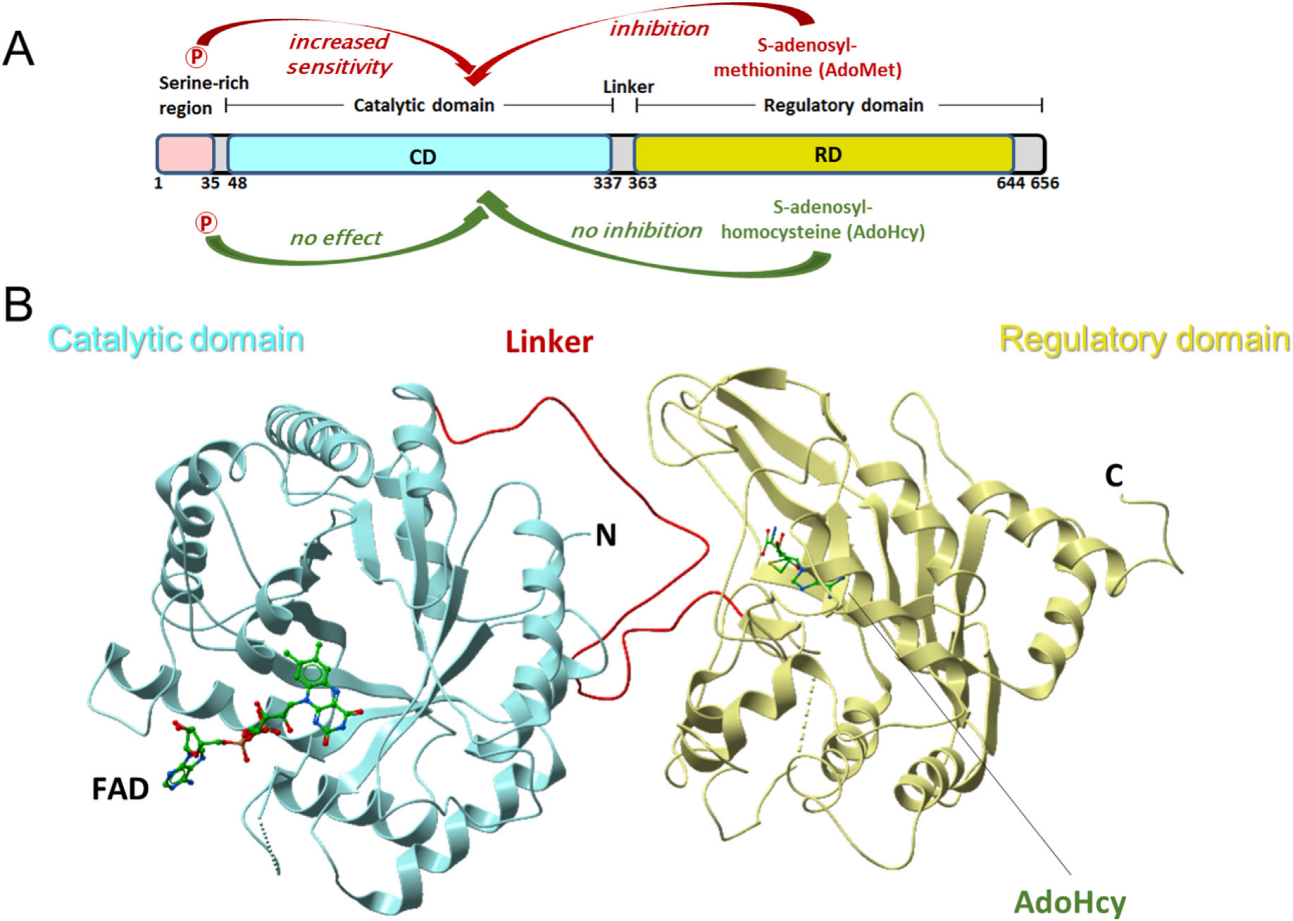
**Schematic and structural representation of human MTHFR. A.** Domain organisation of MTHFR. Numbers given represent approximate amino acid boundaries. **B.** Illustrative structure of MTHFR based on PDB: 6FCX. Domain architecture and bound ligands indicated. FAD: flavin adenine dinucleotide. AdoHcy: *S*-adenosylhomocysteine.

In metazoans, MTHFR activity is regulated by the methionine cycle product AdoMet which acts as an allosteric inhibitor [1] via binding to the C-terminal regulatory domain (RD, Fig. 1A). Phosphorylation of MTHFR, which occurs mainly in the N-terminal serine rich region, further sensitises the protein to this inhibition [[2], [3], [4]]. However, inhibition may be reversed by the binding of s-adenosylhomocysteine (AdoHcy), the de-methylated form of AdoMet, to the RD [5]. We recently determined the crystal structure of human MTHFR from a near-full-length construct [6], showing that the well-conserved catalytic domain (CD) is appended to the RD by an extended linker that traverses between them (Fig. 1B). In our structure, we found AdoHcy bound to the RD, representing the dis-inhibited (hence active) enzyme. Interestingly, structural comparison of the RD, present only in eukaryotic MTHFR, revealed that it represents a new fold distinct from all 18 known classes of AdoMet-binding proteins [7]. AdoMet-binding domains are widespread across evolution, perhaps not surprising given the incredible number of enzymatic reactions dependent on AdoMet as methyl-donor (e.g. ∼2000 AdoMet-dependent methyltransferases in humans [25]). The structural uniqueness of the MTHFR RD presents the opportunity to identify compounds that selectively bind this domain to modulate MTHFR function.

Through modulating the native mechanism of allosteric AdoMet inhibition in two opposite ways, inhibition and dis-inhibition, MTHFR presents a unique point of intervention for diverse diseases associated with OCM dysregulation. Small molecule intervention to dis-inhibit MTHFR, and thereby increase MTHFR activity, could lower the disease burden for severe MTHFR deficiency, a life-threatening metabolic disorder (OMIM #236250; incidence of 1:200,000), characterised by failure to thrive, microcephaly, encephalopathy and seizures [8]. Current mainstay treatment utilises betaine, which provides an alternative substrate to methionine synthesis via betaine-homocysteine S-methyltransferase. However, betaine-homocysteine S-methyltransferase is not present in the brain, and patients often suffer lifelong neurological conditions. The majority of patients harbour missense mutations which reduce, but not ablate, enzymatic activity. While conventional drug development has had limited success with small molecule activators, MTHFR presents a unique opportunity to achieve enzyme activation, by blocking AdoMet-mediated inhibition and therefore dis-inhibiting the enzyme.

On the other hand, dysregulation of OCM in disease states can also be addressed through MTHFR inhibition. Deletion of the *MTHFR* gene has been shown to confer protection against neural tube defects (NTD) and other brain malformations [9,10]. NTDs affect around 1 per 1000 pregnancies worldwide and result from failure in formation of the neural tube. Current treatment involves surgical repair which does not reverse the neurological damage that has already occurred. Primary prevention is the optimal means of therapy. Folic acid supplements can prevent some but not all NTDs (in humans and in mouse models). Moreover, MTHFR has also been proposed as an inhibition target for cancer [11], which heavily utilises OCM. MTHFR expression is significantly upregulated in prostate tumour tissues, correlating with cancer recurrence and death in prostate cancer datasets [12]. Antisense inhibition of MTHFR reduces growth of cancer cell lines in vitro and carcinoma xenografts in vivo [13].

Here, we set out to identify and characterize small molecules with the potential to modulate MTHFR inhibition via the C-terminal RD through a combined approach of biophysical binding assays, enzyme activity assay, and *in silico* docking. Our data revealed a druggable allosteric pocket and a chemical starting point for the development of MTHFR-specific probes to explore its therapeutic potential.

## 2 Material and methods

### 2.1 Expression and purification of recombinant human MTHFR

Cloning, expression and purification of three recombinant human MTHFR constructs, *i.e.* encompassing the full-length (amino acids 1–656; hsMTHFR_FL_), encompassing just the CD and RD (amino acids 38–644; hsMTHFR_CD-RD_), and encompassing the RD and part of the linker region (amino acids 348–656; hsMTHFR_RD_), were carried out as described previously [6].

### 2.2 Enzyme activity assay

All enzymatic assays were performed using the physiological forward assay described by Suormala et al. [14]. For (*S*)-SKI-72 inhibition in HEK239 (ATCC: CRL-3216) cell lysates, the assay was performed with the modifications described by Rummel et al. [15] and Burda et al. [16,17]. For (*S*)-SKI-73 inhibition of intact HEK293 cells, 300,000 cells/well of a 6-well plate were allowed to settle overnight. The next day, media containing Dulbecco’s Modified Eagle Medium (Gibco) supplemented with 10% fetal bovine serum (Gibco) and 1% antibiotic/antimycotic (Gibco) was exchanged to fresh media of the same composition plus the indicated concentration of (*S*)-SKI-73. Following incubation for 12 or 48 h, cells were harvested, lysed and assayed as previously described [15]. Assay of hsMTHFR_FL_ was performed as described [6]. Purified AdoMet was used for inhibition [18]. Sinefungin was purchased from Sigma-Aldrich (Cat# S8559). The *K*_i_ for each compound was estimated following a plot of inhibitor versus response and a four-parameter curve fit as performed by GraphPad Prism (v8.00).

### 2.3 Differential scanning fluorimetry

Differential scanning fluorimetry was used to assay shifts in melting temperature caused by ligand binding in a 96-well PCR plate using an LC480 light cyler (Roche). Each well (20 μl) consisted of hsMTHFR_RD_ protein (0.1 mg ml^−1^), SYPRO-Orange (Invitrogen) diluted 1000X, and buffer (10 mM HEPES pH 7.5, 500 mM NaCl) in the presence of 0–250 μM AdoMet, AdoHcy or analogue compounds. Analogue compounds were part of a AdoMet library consisting of 162 synthetic analogues of adenosine scaffolds which were compiled from the SGC’s compound collection.

### 2.4 MIDAS protein-metabolite interactomics

Protein–metabolite interactomics using an updated MIDAS screening platform was performed as reported previously [19]. Briefly, a flow-injection analysis mass-spectrometry (FIA-MS) method utilising a validated library of 400 metabolite standards was combined into four unique and defined screening pools in 150 mM ammonium acetate pH 7.4. For each metabolite pool, 8 μl of hsMTHFR_CD-RD_ (542 μM) or hsMTHFR_RD_ (240 μM) protein was arrayed in triplicate across a SWISSCI 10 MWC 96-well microdialysis plate (protein chambers). To the trans side of each dialysis well, 300 μl of a metabolite pool (50 μM per metabolite) was arrayed in triplicate for each target protein (metabolite chambers). Dialysis plates were placed in the dark at 4 °C on a rotating shaker (150 rev min−1) and incubated for 40 h. Post-dialysis, protein- and metabolite-chamber dialysates were normalized and diluted 1:10 in 80% methanol, incubated for 30 min on ice, and centrifuged at 3200 RCF for 15 min to remove precipitated protein. Metabolite pool analytes were arrayed across a 384-well microvolume plate and placed at 4 °C in a Shimadzu SIL-20ACXR autosampler for FIA-MS metabolomics analysis. 2 μl of each sample was analysed in technical triplicate by FIA-MS on a SCIEX X500R QTOF MS with interspersed injections of blanks.

### 2.5 MIDAS data analysis

FIA-MS spectra from MIDAS protein–metabolite interactomics was qualitatively and quantitatively processed in SCIEX OS 1.6 software to determine relative metabolite abundance by integrating the mean area under the extracted-ion chromatogram curve across technical triplicates. Log2 (fold change) for each metabolite was calculated from the relative metabolite abundance in the protein chamber (numerator) and metabolite chamber (denominator) from dialysis triplicates. For each technical triplicate, up to one outlier was removed using a z-score cutoff of five (<0.1% of observations). The corrected technical replicates were collapsed to one mean fold-change summary per protein–metabolite pair. To remove fold-change variation that was not specific to a given metabolite–protein pair, the first three principal components of the total MIDAS dataset were removed (∼75% of observed variance) creating Log2 (corrected fold change). Protein–metabolite z-scores were determined by comparing the target protein–metabolite Log2 (corrected fold change) to a no-signal model for that metabolite using measures of the central tendency (median) and standard deviation (extrapolated from the 25–75% quantiles) which are not biased by the signals in the tails of a metabolite’s fold-change distribution. Z-scores were controlled for false-discovery rate using Storey’s q value (http://github.com/jdstorey/qvalue). Protein–metabolite interactions with p-values < 0.05 and q-values < 0.1 were considered significant.

### 2.6 Surface plasmon resonance (SPR)

For determination of the affinity between hsMTHFR_CD-RD_ and (S)-SKI-72, 30 μg of hsMTHFR_CD-RD_ was immobilized onto Sensor Chip CM5 sensor (series S) to a density of 7500 RU via the protein –NH_2_ groups employing 1-Ethyl-3-(3-dimethylaminoproppyl)carbodiimide crosslinker (EDC) and N-hydroxysuccinimide (NHS) in acetate buffer pH 4.0. For the binding experiment, (S)-SKI-72 was serially diluted (1:1) from 6.25 μM to 0.05 μM in 20 mM HEPES pH 7.5, 150 mM NaCl, 0.5 mM TCEP, 0.05% TWEEN-20, 5% DMSO and subsequently injected at a 30 μL/min flow rate over the sensor. For determination of the affinity of hsMTHFR_RD_ for (*S*)-SKI-72 and AdoMet, 30 μg of hsMTHFR_RD_ was immobilized onto a Ni-NTA sensor via the protein His-tag to a density of 3000 RU and 2550 RU. For the binding experiment, (S)-SKI-72 was serially diluted from 12.5 μM to 0.012 μM and AdoMet was serially diluted from 25 μM to 0.19 μM and subsequently injected at a 30 μL/min flow rate over the sensor. Data were analysed using the Biacore S200 Evaluation Software.

### 2.7 Docking

The 3D chemical structures of TAM-4-61, TAM-4-59, WZ-16 (6′-homosinefungin), sinefungin, and *(S)*-SKI-72 were prepared using Ligprep of Schrodinger (version 2020–3). The protonation state of each ligand was determined by Epik of Schrodinger (version 2020–3) in pH 7.0 ± 2.0 condition. The structure of hsMTHFR was obtained from the Protein Data Bank (PDB code 6FCX). In our induced fit docking (IFD) [20] studies, we assumed the adenine and tetrahydrofuran-3,4-diol moieties common to all ligands should be bound in the same binding pocket, and applied core-constraints with a tolerance of 1.0 Å. As a control, we also docked AdoHcy back to the crystal structure (PDB code 6FCX), and noted the most dominant pose is the one found in the original structure.

## 3 Results & discussion

### 3.1 Identification of compounds bound by the regulatory domain of MTHFR

**Fig. 2:**
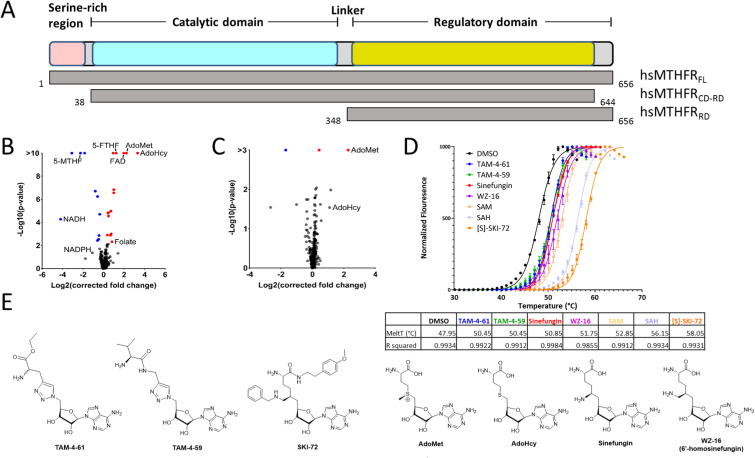
**Identification of compounds bound by the regulatory domain of MTHFR. A.** Schematic domain organisation of MTHFR depicted above in colour with protein constructs used in this study visualized below in grey. Amino acid boundaries for each are given, based on NP_005948. **B.** The hsMTHFR_CD-RD_–metabolite interactome as determined by MIDAS. AdoMet, AdoHcy, FAD, folate, and 5-FTHF were enriched and 5-MTHF, NADH, and NADPH were depleted. **C.** The hsMTHFR_RD_–metabolite interactome as determined by MIDAS. AdoMet and AdoHcy were enriched. B and C, red data points indicate significantly enriched metabolites and blue data points indicate significantly depleted metabolites. The cut-off for significance was p < 0.05 and q < 0.1. **D.** Above: Representative curves of the differential scanning fluorimetry binding assay for hsMTHFR_RD_ in the absence (DMSO) or presence of 250 μM of each compound. Each curve represents *n* = 2, replicates pooled and fitted. Below: Table indicating the average melting point and goodness of fit for each curve. **E.** Structures of the compounds indicated in panel D.

We explored the ligand binding specificity of MTHFR using a mass spectrometry-based equilibrium dialysis protein-metabolite interactomics approach (MIDAS) to reveal non-catalytic and catalytic protein-metabolite interactions [19]. Multiplexed screening of a library of 400 human metabolites was carried out against a near full-length construct of MTHFR containing the CD and RD but lacking the N- and C-termini (hsMTHFR_CD-RD_, Fig. 2A). This revealed detectable interactions with native ligands including the substrate 5-methyltetrahydrofolate (5-MTHF; *p <* 1.57 × 10^−45^, *q <* 8.04 × 10^−43^), substrate analogue folate (*p <* 4.85 × 10^−3^, *q <* 8.07 × 10^−2^), co-factors NADH (*p <* 5.63 × 10^−5^, *q <* 1.99 × 10^−3^), NADPH (*p <* 4.36 × 10^−2^, *q <* 3.59 × 10^−1^), and FAD (*p <* 2.48 × 10^−38^, *q <* 1.02 × 10^−35^), allosteric regulators AdoMet (*p <* 1.18 × 10^−15^, *q <* x1.95 × 10^−13^) and AdoHcy (*p <* 3.39 × 10^−14^, *q <* 4.98 × 10^−12^), and the inhibitor 5-formyltetrahydrofolate (5-FTHF; *p <* 3.59 × 10^−15^, *q <* 5.80 × 10^−13^) (Fig. 2B). The negative fold changes observed for 5-MTHF, NADH, and NADPH suggest that hsMTHFR_CD-RD_ was catalytically active during the MIDAS assay. Importantly, when screening against the RD alone (hsMTHFR_RD_, Fig. 2A) we observed interactions with AdoMet (*p <* x3.2810^−16^, *q <* 5.68 × 10^−14^) and AdoHcy (*p <* 2.89 × 10^−2^, *q <* 2.79 × 10^−1^), but not the substrate and cofactors (Fig. 2C). These data indicate that interactions with substrate and cofactor require the catalytic domain, while the RD is sufficient to bind AdoMet/AdoHcy.

Taking advantage that the RD represents a novel AdoMet-binding fold [6] and offers opportunities for designing specific binders, we set out to explore its druggability through small molecule screening. Using differential scanning fluorimetry (DSF), we screened a library of 162 AdoMet mimetics for binding towards hsMTHFR_RD_, to be detected through changes in the protein melting temperature (T_*m*_). We observed 4 novel compounds which exhibited a significant increase in thermostability when incubated at concentrations of up to 250 μM with 0.1 mg ml^−1^ hsMTHFR_RD_ (Fig. 2D). Three of these compounds, TAM-4-61, TAM-4-59 and WZ-16 (6′-homosinefungin) had T_*m*_ shifts of approximately 3–4 °C (Fig. 2D). They structurally resemble the pan-methyltransferase inhibitor sinefungin (Fig. 2E), which exhibits a similar T_*m*_ shift (Fig. 2D). Derivatives of sinefungin have found diverse application as anti-fungal [21], anti-viral [22] and anti-cancer [23]. The native ligands of MTHFR, AdoMet and AdoHcy, induced T_m_ shifts of approximately 4 °C and 7 °C, respectively (Fig. 2D). Notably, the fourth and chemically distinct compound, (S)-SKI-72, showed the greatest increase of stability, with a T_m_ shift of approximately 10 °C (Fig. 2D). (S)-SKI-72 was previously developed as a potent inhibitor of the protein arginine methyltransferase 4 (PRMT4) [24]. Compared to sinefungin and AdoHcy/AdoMet, (*S*)-SKI-72 is further derivatised with an *N*-benzyl substituent at the 6′ position and substitution at the α-amino carboxylate moiety (Fig. 2E).

### 3.2 (*S*)-SKI-72 is bound by MTHFR in solution

**Fig. 3:**
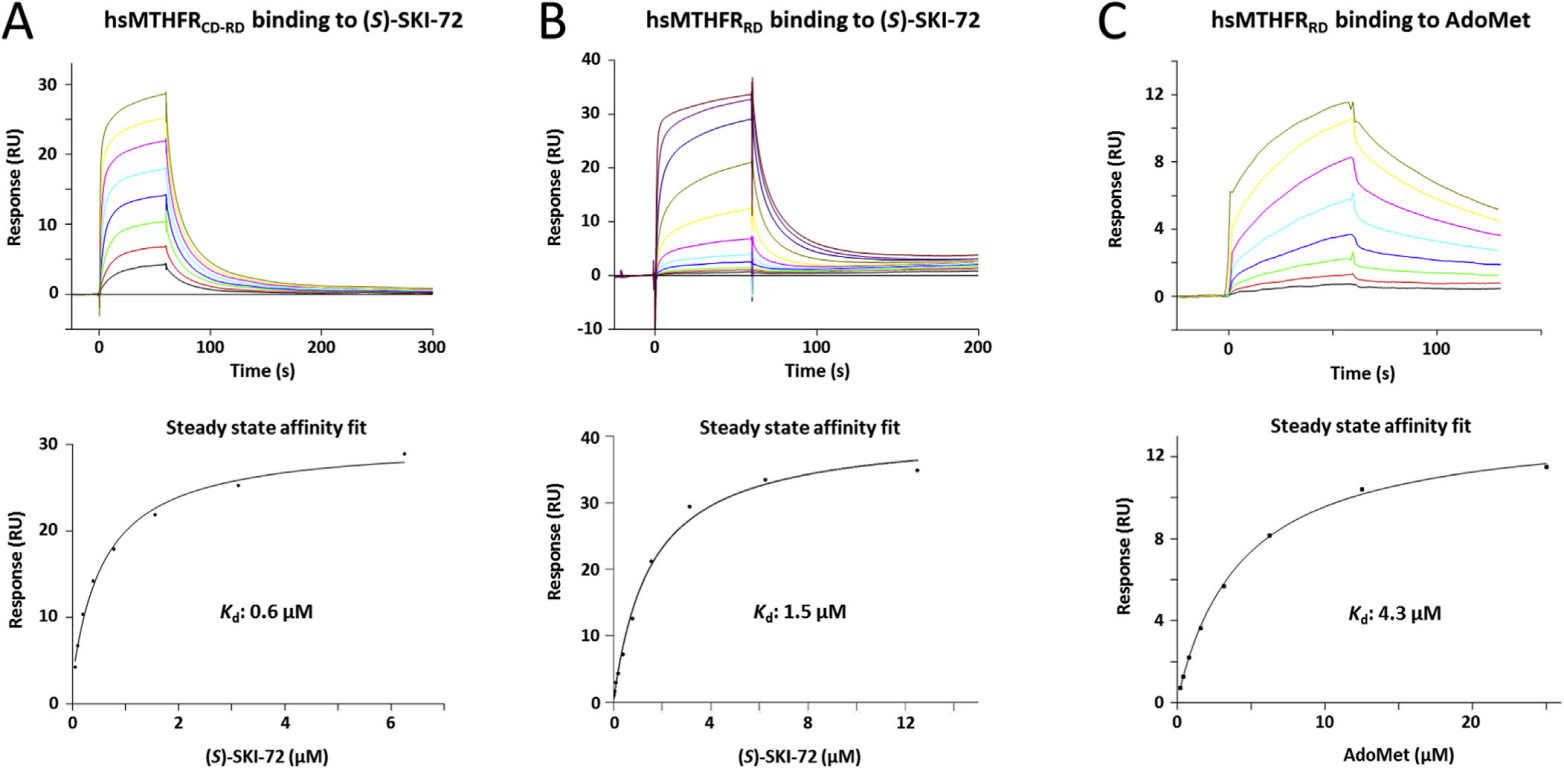
**Characterization of hsMTHFR**_**RD-CD**_**and hsMTHFR**_**RD**_**binding to (*S*)-SKI-72 and/or AdoMet via SPR. A-C.** Above: Representative sensorgram plots of response units (RU) against time for different concentrations of the ligands. Below: sensorgram plots of response against ligand concentration. Data were fitted using steady state affinity equation. Binding affinity indicated by the dissociation constant (*K*_d_). Each curve is a representative of *n* = 2 replicates. Complete data: hsMTHFR_CD-RD_ binds (*S*)-SKI-72: *K*_d_ = 596 nM and 612 nM hsMTHFR_RD_ binds (*S*)-SKI-72: *K*_d_ = 1.47 μM and 1.175 μM hsMTHFR_RD_ binds AdoMet: *K*_d_ = 1.057 μM and 4.3 μM.

Since (*S*)-SKI-72 is a much larger scaffold than the others, and structurally distinct from AdoMet/AdoHcy, we leveraged an orthogonal in vitro assay to validate and quantify its binding to both hsMTHFR_CD-RD_ and hsMTHFR_RD_. Employing surface plasmon resonance (SPR), we verified that hsMTHFR_CD-RD_ binds (*S*)-SKI-72 with sub-micromolar affinity (dissociation constant, *K*_d_: 612 nM) (Fig. 3A). Similarly, hsMTHFR_RD_ binds (*S*)-SKI-72 with micromolar affinity (*K*_d_: 1.47 μM) (Fig. 3B). This is comparable to the measured affinity of hsMTHFR_RD_ for its native ligand AdoMet (*K*_d_: 4.3 μM) (Fig. 3C). These data suggest (*S*)-SKI-72 to be avidly bound by the RD of MTHFR.

### 3.3 Flexibility of the regulatory domain to accommodate ligands

**Fig. 4:**
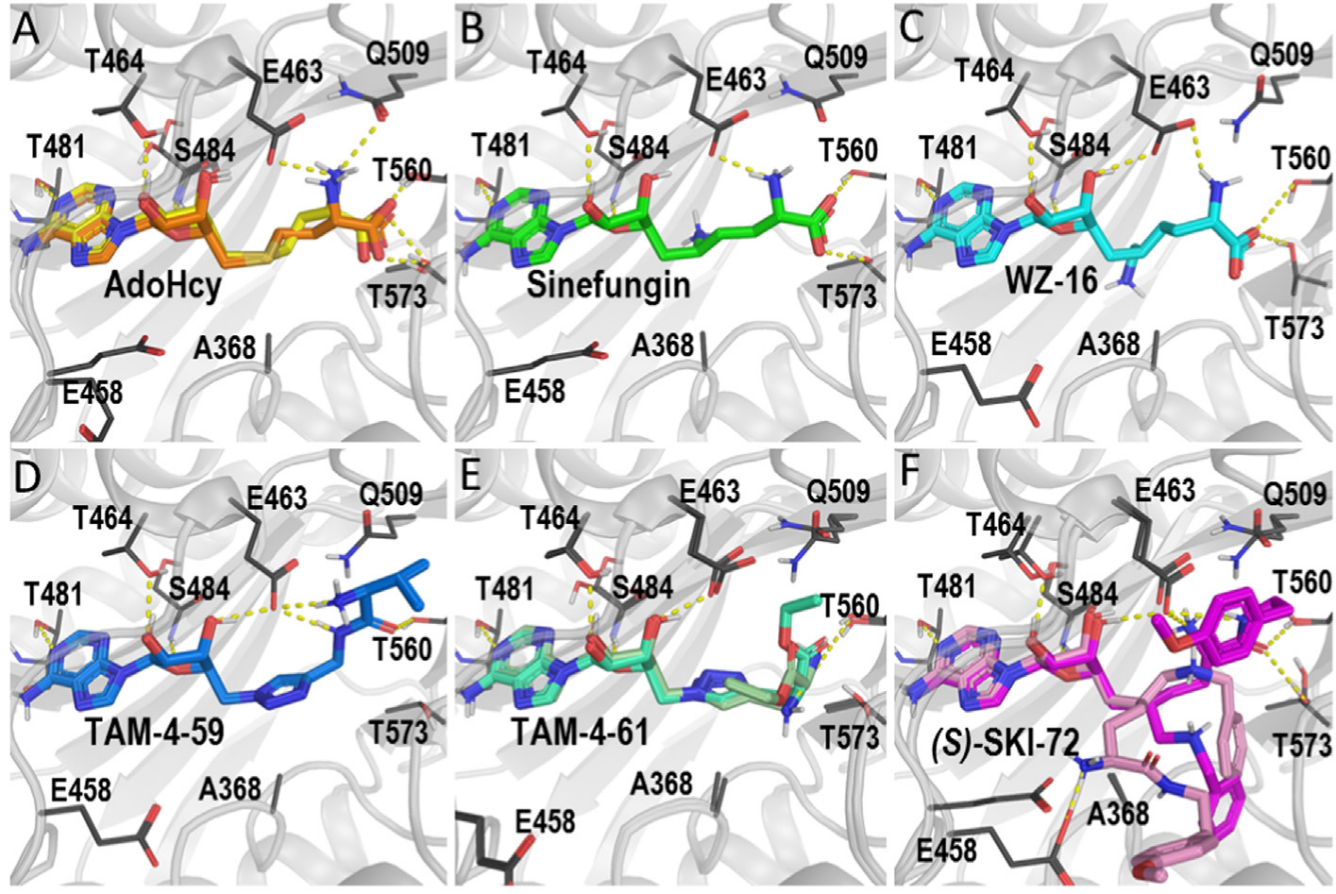
***(S)*-SKI-72 has an alternative binding pose compared to sinefungin and its analogues. A.** The AdoHcy pose found in the crystal structure is in orange, the docking pose is in yellow. **B-E**. Sinefungin and its analogues show similar poses in the binding site. In panel E, the *S*-isoform of TAM-4-61 is coloured as greencyan, while its *R*-isoform is in palegreen. **F.** Two possible poses of (*S*)-SKI-72, one (darkpink) is similar to those shown in A-E, and the other (palepink) forms an ionic interaction with Glu458.

To characterize the binding poses of ligand hits, we performed molecular docking of these compounds in the RD of our hsMTHFR_CD-RD_ structure originally bound with AdoHcy (PDB code 6FCX). Our results showed that sinefungin and its analogues have similar binding poses to that of AdoHcy found in the crystal structure. Thr464 and Glu463 from the loop segment preceding α5 of the MTHFR RD, Ser484 and Thr481 from the central β-sheet strand β10, and Thr560 from strand β16, interacted with all of these ligands (Fig. 4A–F). Besides these five residues, Gln509 and Thr573 form polar interactions with AdoHcy, which may contribute to its higher affinity than TAM-4-59 and TAM-4-61 (Figs. 2D and 4A, D and E). For WZ-16 and sinefungin, Thr573 interacts with their carboxy groups at the tail of the ligands and further stabilizes the poses (Fig. 4B and C).

Interestingly, we found that *(S)-*SKI-72 can be in two poses hinged around the chiral 5-position of the hexanamide. One pose is very similar to those of sinefungin and its analogues as described above. Alternatively, *(S)-*SKI-72 can adopt a different pose by forming a salt bridge with Glu458 and a hydrophobic interaction with Ala368 (Fig. 4F). Such a divergent pose may contribute to the different pharmacological properties of *(S)-*SKI-72.

### 3.4 Different effects of (*S*)-SKI-72 and sinefungin on MTHFR activity

**Fig. 5:**
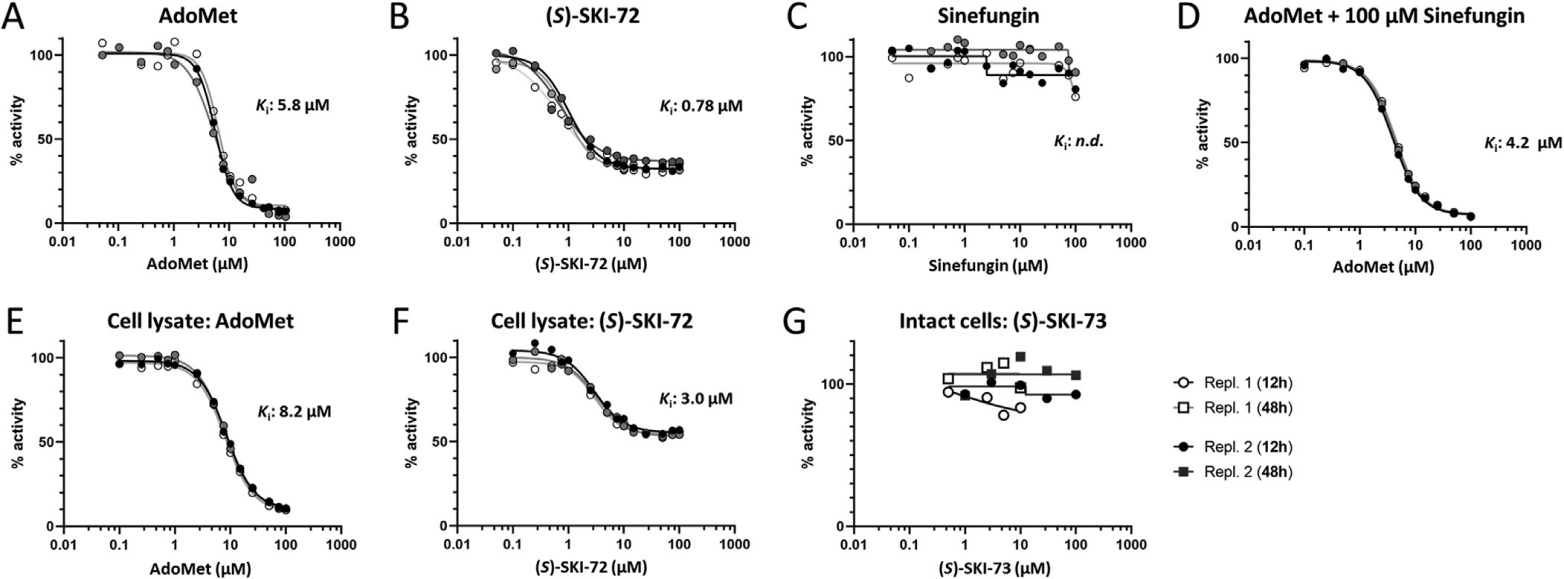
**Assay of MTHFR activity following addition of AdoMet, (S)-SKI-72 or sinefungin. A-C.** Remaining activity of hsMTHFR_FL_ following incubation with increasing concentrations of AdoMet (A), (*S*)-SKI-72 (B) or sinefungin (C). **D.** Remaining activity of hsMTHFR_FL_ with increasing concentrations of AdoMet following pre-incubation with 100 μM sinefungin. **E-F.** Remaining activity of endogenous MTHFR from HEK293 cell lysate following incubation with increasing concentrations of AdoMet (E) or (*S*)-SKI-72 (F). **G.** Remaining activity of endogenous MTHFR from HEK293 cell lysate following incubation of (*S*)-SKI-73 with live cells for 12 or 48 h. Repl: replicate. Each curve represents a single technical replicate, of which *n* = 3 were performed in each assay. Curve was fit and inhibitor constant (*K*_i_) calculated as described in the Materials and Methods.

Finally, we examined the effect of (*S*)-SKI-72 and sinefungin (as an archetype of WZ-16, TAM-4-59 and TAM-4-61) on the activity of MTHFR. Two enzymatic outcomes are possible with modulators of hsMTHFR_RD_. Like its native ligand AdoMet, ligands targeting the RD may result in partial or complete enzymatic inhibition. Alternatively, as is the case with AdoHcy, the demethylated metabolite of AdoMet, ligands binding to the RD may lock the enzyme into the active, or dis-inhibited, state. Through modulation of enzyme activity in these two opposite ways, MTHFR presents a unique point of intervention for diverse diseases associated with one carbon metabolism. Using purified hsMTHFR_FL_ as a positive control, we identified AdoMet to inhibit MTHFR with a *K*_i_ of 5.8 ± 0.2 μM (Fig. 5A), comparable to the previously published value [6]. (S)-SKI-72 was found to be a more potent inhibitor of hsMTHFR_FL_ than AdoMet, with a K_i_ of 0.78 ± 0.05 μM (Fig. 5B). This is consistent with its K_d_ of 0.6 nM (Fig. 3A) and reflective of its higher affinity to hsMTHFR_RD_ compared to AdoMet (Fig. 3B and C). Surprisingly, unlike AdoMet, (*S*)-SKI-72 was unable to fully inhibit hsMTHFR_FL_, which retained a residual activity of ∼30% even at high concentrations (Fig. 5B). Altogether, (*S*)-SKI-72 and its derivatives could be developed to lock MTHFR in an inhibited state.

On the contrary, sinefungin had no effect on the activity of hsMTHFR_FL_ at concentrations up to ∼100 μM, after which it appeared to inhibit the enzyme (Fig. 5C). The ability to inhibit hsMTHFR_FL_ at very high concentrations was confirmed by assay of increasing concentrations of AdoMet (0.05–100 μM) in the presence of 100 μM sinefungin. This resulted in a *K*_i_ of 4.2 ± 0.1 μM (Fig. 5D), which was slightly reduced compared to AdoMet alone (Fig. 5A). Therefore, sinefungin and its derivatives could be developed into a dis-inhibitor of MTHFR.

To assess the potential utility of (*S*)-SKI-72 as a modulator of MTHFR, we investigated its effect on the activity of endogenous MTHFR from HEK293T cell lysates. As a positive control, we found application of AdoMet to cell lysates to inhibit endogenous MTHFR with a *K*_i_ of 6.8 ± 0.2 μM (Fig. 5E). In this milieu, (*S*)-SKI-72 inhibited endogenous MTHFR with a *K*_i_ of 3.0 ± 0.2 μM (Fig. 5F). Once again, (*S*)-SKI-72 was more potent than AdoMet but unable to fully inhibit MTHFR, whereby approximately 50% residual activity remained even at the highest concentrations tested (Fig. 5F).

Finally, since (*S*)-SKI-72 exhibits poor membrane permeability [24], we examined the effect of its prodrug derivative (S)-SKI-73, in which the 9′-amine moiety is cloaked with the trimethyl-locked quinone butanoate moiety, on intact HEK293 cells. Previous observations have shown that once (S)-SKI-73 passes inside the cell membrane, it is metabolised into (S)-SKI-72 and 6′-N-benzyl-homosinefungin, which then accumulates inside the cell [24]. Following 12 or 48 h incubation of (S)-SKI-73 added to the media of HEK293 cells, assay of MTHFR activity from cell lysates revealed no effect on residual MTHFR activity regardless of the concentration of (S)-SKI-73 provided (Fig. 5G). The underlying basis for this lack of effect can be due to the predominant conversion of (S)-SKI-73 into 6′-N-benzyl-homosinefungin rather than (S)-SKI-72 inside living cells [19].

## 4 Conclusion

The two-domain architecture of hsMTHFR implies that AdoMet mediates its inhibitory effect through a long-range conformational change in order to transmit the AdoMet-bound signal from the RD to the active site in the CD through the extensive inter-domain linker. The outcome of the signal transduction event could be occlusion of the CD active site, thereby inhibiting MTHFR activity. Our data here point to two non-mutually exclusive speculations on why (S)-SKI-72, which appears to interact extensively with hsMTHFR through the RD, may have been a more potent inhibitor of MTHFR than AdoMet, but was unable to fully inhibit the enzyme. First, the underlying conformational change following (S)-SKI-72 binding may not occlude the CD to the same extent as does AdoMet, due to subtle differences in the binding modes between (S)-SKI-72 and AdoMet. Second, the two distinct binding poses of (S)-SKI-72 identified by docking, one adopting an orientation similar to the non-inhibiting sinefungin and the other a likely inhibitory pose as suggested by its interaction with Ala368, may imply heterogeneity that complicates the response towards enzyme activity by the compound. Future investigation of conformational flexibility and protein dynamics by experimental (e.g. cryo-electron microscopy) and computational (molecular dynamics simulation) approaches are clearly merited.

## Author contributions

M.L., L.S., D.S.F. and W.W.Y. contributed to the conception of the work, G.A.B., A.H., W.R.F., B.X., K.G.H., C.B., S.L., A.M., D.S. and D.B. contributed to the collection of data, G.A.B., A.H., B.X., K.G.H., J.R., A.T., P.J.B., M.L., L.S., D.S.F. and W.W.Y. contributed to data analysis, M.L., L.S., D.S.F. and W.Y.Y. wrote the manuscript with contributions from all the other authors.

All authors have approved the final article.
